# At a London Children's Hospital

**Published:** 1913-01-04

**Authors:** 


### AT A LONDON CHILDREN'S HOSPITAL.

### A Doctor Illustrates His Hobby.

The wards of the Paddington Green Children's Hospital were decorated by the Matron, Miss E. Sheriff-MacGregor, together with the sisters and the nurses. A large Christmas tree was presented by Mr. Walter G. Howes, gay with the gilt flowers of electric light, and loaded with toys, the gifts of other friends, which vied with the lights as the chief attraction for the children. The Chairman of the Hospital, Mr. Douglas Owen, Mr. Lionel Hanbury, the Editor of *Truth*, and numerous other supporters of the institution have sent gifts of games, toys, and clothing. On Christmas morning each of our patients received a bright new shilling and a bag well filled with toys, which "Santa Claus" hung upon their cots.

The usual Christmas fare was also enjoyed on the Day, but the Christmas entertainments were not given till Friday, December 27, when, from 3 to 5 o'clock P.M., a lantern display kindly provided by Dr. Herbert E. Friend was the chief event of the entertainment. Various views and pictures illustrated the life of the hospital wards, reproduced the tile picture panels in the new out-patient waiting hall, and were descriptive of Paddington, past and present, on which Dr. Friend is probably a unique authority, for his collection of old maps and prints of the district is very large, and its gathering has been the chief relaxation of his leisure. But the lantern did not forget the illustrative possibilities of nursery stories either. The toys, dolls, and games from the Christmas tree were distributed among the patients by members of the committee of management and the medical staff.

